# Effect of Aluminum Flakes on Mechanical and Optical Properties of Foam Injection Molded Parts

**DOI:** 10.3390/polym13172930

**Published:** 2021-08-30

**Authors:** Donghwi Kim, Youngjae Ryu, Ju-Heon Lee, Sung Woon Cha

**Affiliations:** Department of Mechanical Engineering, Yonsei University, 50 Yonsei-ro, Seodaemun-gu, Seoul 03722, Korea; donghwi.kim@yonsei.ac.kr (D.K.); yjryu1027@yonsei.ac.kr (Y.R.); wngjs1009@yonsei.ac.kr (J.-H.L.)

**Keywords:** aluminum flakes, metallic texture, conventional injection molding, foam injection molding, reflectance, gloss, mechanical properties

## Abstract

Injection research using aluminum flakes has been conducted to realize metallic textures on the surface of plastic products. Several studies have focused on the effect of the orientation and quality of the flakes when using conventional injection molding methods; however, limited studies have focused on the foam injection molding method. In this study, we examined the orientation of aluminum flakes through foam injection with an inert gas and observed the changes in texture using a spectrophotometer and a gloss meter. The mechanical properties were also studied because the rigidity of the product, which is affected by the weight reduction that occurs during foaming, is an important factor. The results demonstrate that under foam injection molding, reflectance and gloss increased by 6% and 7 GU, respectively, compared to those obtained using conventional injection molding; furthermore, impact strength and flexural modulus increased by 62% and 15%, respectively. The results of this research can be applied to incorporate esthetic improvements to products and to develop functional parts.

## 1. Introduction

The appearance of plastic products is crucial in satisfying the purchasing needs and sensibilities of consumers [1]. Recently, visual upgrades have been applied for various colors, and as an increasing number of customers are looking for deep colors, manufacturers are focusing on developing aesthetic designs for their products [2]. To create these aesthetic designs, additional processing such as coating and plating is carried out on the surface to provide a metal texture that evokes depth [3]. Reflectivity is important for creating metal textures [4,5]. However, adding a coating to the plastic molding increases either costs or production time, which results in significant economic losses for the manufacturer. In addition, the coating does not allow for even coloring, and the high incidence of volatile organic compounds causes many environmental pollution problems [6,7]. To solve these problems, methods of adding metal pigments to thermoplastics have been extensively studied [8,9,10,11,12,13,14,15,16,17,18]. Based on previous research, we used various metal pigments or aluminum flakes to address the effects of the orientation of the aluminum flakes on appearance [8,9,10,11,12,13,14,15,16,17,18]. Aluminum fragments are broad plate shapes with different orientations from fiber, and the Jeffrey and Folgar–Tucker models can be used to predict these fiber orientations and conduct theoretical and quantitative investigations [8,9,11,12,13,14,15,16]. In addition, the orientation of aluminum flakes causes problems such as the well-line, which has been described in a study on the differences in flow lines and flake orientation [10,17,18]. Interest in aluminum flakes persists, and according to TechNavio Research, the market size of aluminum paste/flakes is expected to expand to around 120,000 tons between 2021 and 2025, with a compound annual growth rate of 5% [19].

In this study, we examined the changes in the reflectivity of metals by injecting gas into existing aluminum flake injections. The injection of inert gases is called foam injection molding (FIM), which requires gas injection devices, in contrast with conventional injection molding (CIM). The main difference between CIM and FIM is that supercritical carbon dioxide or nitrogen is introduced in the barrel of the injection machine during the plasticizing process in FIM, and the injected gas assumes a single liquid form with the molten resin [20,21,22]. When the gas is saturated at high pressure, the pressure drops sharply as the resin is injected into the mold, and foaming occurs [20,21,22]. FIM creates a partially hollow part due to gas injection, which can save 10–20% of the resin material. Moreover, the cycle time is shortened owing to the replacement of the holding pressure process of the conventional injection method [23,24,25]. In addition, because of the bubbles inside the molded product, the weight is reduced and the impact strength is increased [26]. Furthermore, research has demonstrated that reflective performance can be improved when porosity is induced through foam, allowing the production of highly reflective and lightweight products [27].

To date, no studies have focused on aluminum-added FIM. A metallic texture can be attained without the painting process by adding aluminum flakes in CIM. However, FIM can improve reflectance and reduce weight while maintaining and strengthening the metal texture. The weight reduction and appearance of plastic products are major issues in the automobile and mobile phone industries, and through this study, these aspects can be dealt with adequately according to consumers’ needs [28]. These results were evaluated with optical equipment, and if aluminum foam injection products are diversified, the findings of this study can be applied in other fields, such as for electromagnetic shielding and heat transfer media.

## 2. Materials and Methods

### 2.1. Materials

#### 2.1.1. Base Resin

PP (Lotte Chemical Corp., Seoul, Korea) was used as the synthetic resin in the injection experiments. PP, a representative general-purpose plastic, was selected because it is transparent and allows for the observation of aluminum flakes. The heat deflection temperature and melting index were 120 °C and 10 g/10 min, respectively.

#### 2.1.2. Pigments

In this study, aluminum flakes from Silberline (Tamaqua, PA, USA) were used to produce the metallic textures. Aluminum produced in pellet form comprises various graded particles (Table 1), which exhibit different textures depending on the type, size, and content of the pigment.

#### 2.1.3. Foam Gas

The gas used for FIM was nitrogen (Purity 99.9%, 40 L, Samhung GasTech, Seoul, Korea). The critical temperature and critical pressure of nitrogen are 126.19 K and 34 bar, respectively, and nitrogen can be made to exhibit a supercritical state more easily than carbon dioxide [29]. It can be used as a physical foaming agent to produce stable microcellular foam and was applied to a resin with added aluminum flakes.

### 2.2. Methods

#### 2.2.1. Processing and Foaming

**Figure 1:**
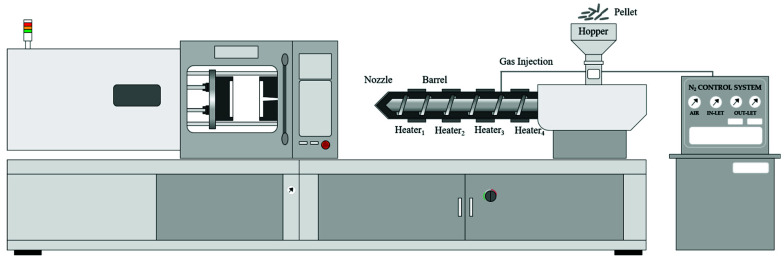
Schematic of injection molding machine of conventional and foam injection method.

All experiments were conducted using a syringe (SELEX-E120, Woojin Plaimm Corp., Boeun-gun, Korea). The injection molding machine uses a double toggle method, comprising a screw diameter of 40Φ, L/D ratio of 20:1, and compression ratio of 1.87:1. During the molding process, the aluminum pellets were mixed, and the resin and mixed materials were transferred into hoppers. The mixing resin was plasticized by the rotation of the barrel heater and screw of the ejector and was then injected into the mold through the nozzle, and the sample was taken through to the cooling stage (Figure 1).

Two injection methods were used: the conventional injection method and foam injection method. The conventional injection proceeded by the same steps as above, and foam injection was performed by injecting supercritical nitrogen into the barrel at the feeding stage. Using these methods, we first examined how the mold differs according to the type, size, and content of aluminum particles used during the CIM and FIM processes, based on a graded optimized metal texture. The injection temperature and mold temperature are higher than usual because lower temperatures coagulate the polymer faster, resulting in more visual defects, such as the weld line (Table 2) [30].

**Figure 2:**
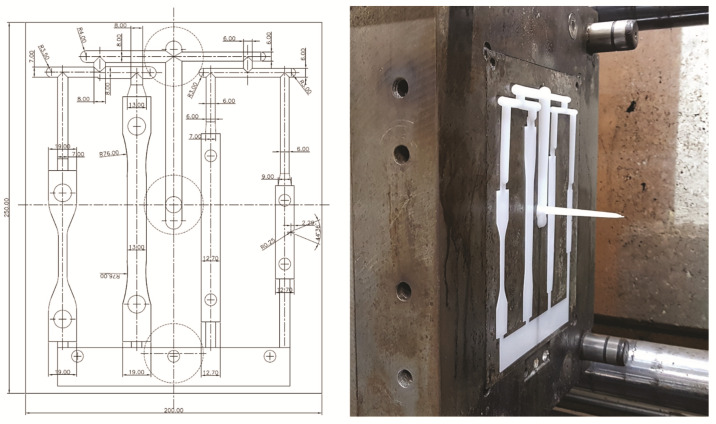
Injection molding ASTM standard test mold.

**Figure 3:**
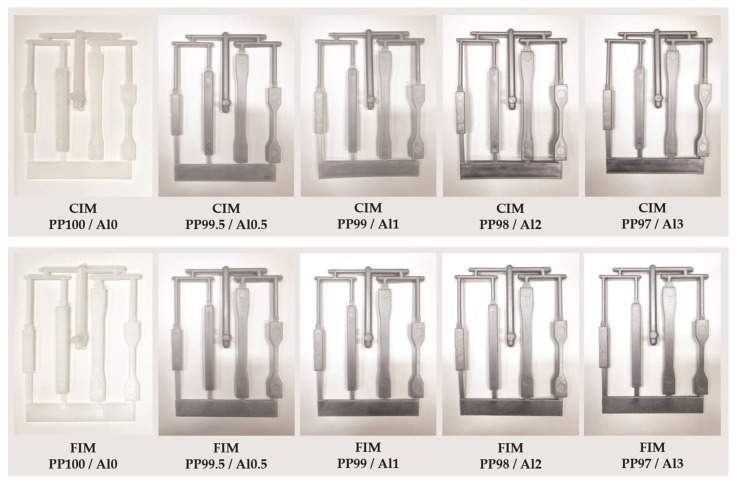
Appearance of conventional and foam injection specimens with different aluminum contents.

The mold used consists of a shape that can identify the tensile, flexural, and impact properties of the used plastic (Figure 2), and the optical and mechanical properties of the final molded specimen were examined (Figure 3).

#### 2.2.2. Measurement

Density is the most important factor for determining the foaming rate of a specimen. It was measured using a density meter (MD-300S, Alfa Mirage, Osaka, Japan). The weight was measured using an underwater substitution method based on an electronic scale, and the minimum unit is displayed up to 0.001 g/cm^3^. The foaming ratio was calculated using the weight calculated using Equation (1) [31]:

Foaming ratio (%) = (D_0_ − D_f_)/D_0_, (1)

where D_0_ is the density of the polymer before foaming and D_f_ is the density of the polymer after foaming.

For the optical analysis, we largely employed a spectrophotometric colorimeter and gloss meter. Using the spectrophotometer (CM-3500D, Konica Minolta, Tokyo, Japan), the reflection spectrum was collected at wavelengths of 360 to 740 nm in units of 10 nm, and the chromaticity (CIE L*, a*, b*) was evaluated.

Gloss measurements were taken using a gloss meter (MG268-F2, KSJ, China) that was compatible with the following test methods: ASTM D2457 and ASTM D523. Seven tensile specimen surfaces were measured at various incident angles (20°, 60°, and 85°), with the average luster represented in terms of GU.

A field-emission scanning electron microscope (JSM-7001F, JEOL Ltd., Japan) was used to investigate the orientation of the aluminum contained in the specimen. To create a smooth cross section, the tensile specimen was rapidly frozen and broken using liquid nitrogen, and the severed specimen was fixed in the cross-section holder. The shape was then observed using a scanning electron microscope (SEM) under vacuum (10^5^ Pa) and 15 kV accelerated voltage after 100 s of Pt coating with ion sputtering (108 auto, Cressington Ltd., Dortmund, Germany) to prevent charge-up phenomena in non-conductive materials.

Material measurements were made using a QM100T, QMESYS Co. Ltd., Gunpo-si, Korea, and digital Izod impact tests (CKII-910D, CKSI, Suwon-si, Korea). The tensile strength and flexural modulus were measured at speeds of 40 and 20 mm/min, respectively, in accordance with ASTM D638 and ASTM D790. The impact strength was measured by calculating the energy required to destroy the specimen at a lifting angle of 150° and an impact velocity of 3.46 m/s.

## 3. Results and Discussion

### 3.1. Appearance Evaluation

#### 3.1.1. Metallic Texture Resulting from Aluminum Particle Addition

Various factors determine the metallic texture of the specimen to which aluminum flakes are added. One of them is luminance, which refers to the degree of sparkle and gloss [32]. Luminance is the degree of brightness projected from the light source and is denoted by L*. The aluminum particles used in this study are in the shape of silver dollars and cornflakes. When compared with particles of similar content and size, silver dollars are known to have higher luminance (Table 3).

**Figure 4:**
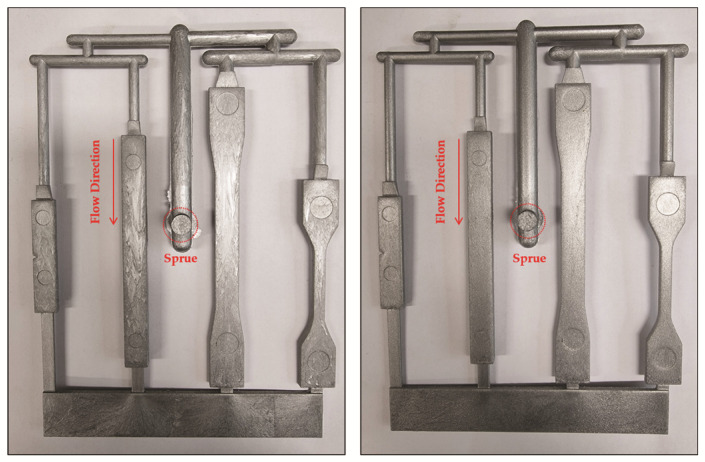
Comparison of the appearance of foam injection products produced with different sized of aluminum flakes: 13 μm (**left**), 65 μm (**right**).

Silver dollars are rounded flat disks, from which light is reflected in opposite directions, while the cornflake pigment has irregular ends, which reduces its luminance [33]. In addition to the type, the size of aluminum particles also influenced the luminance, because small particles are more diffuse than large particles (Table 4). However, a higher dispersion indicated that a greater number of surface defects, such as gas flow marks, were present (Figure 4).

The increased aluminum flake content improved the texture. However, considering the slight increase and high cost of the additives, up to 3 wt% was confirmed to be optimal (Table 5).

#### 3.1.2. Reflectance and Gloss of CIM and FIM

**Figure 5:**
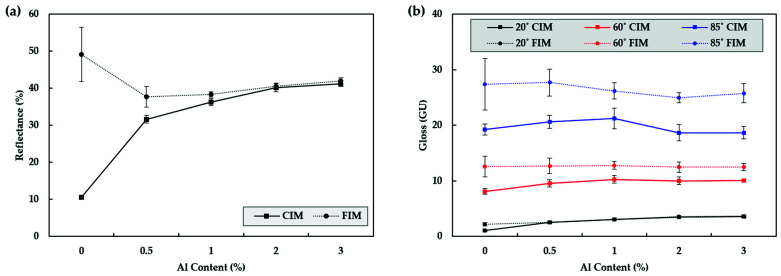
(**a**) Reflectance and (**b**) gloss for products produced using CIM and FIM with different quantities of aluminum.

In both the conventional injection and foam injection methods, the aluminum content was increased up to 3% by adding 1%. In the case of reflectance, the lower was the content, the greater was the difference between the CIM and FIM results, so additional 0.5% content was also produced and examined. To calculate the reflexivity, data from the bite and parallel parts of seven specimens was averaged, and the gloss was averaged from seven specimens, with three measurements taken from the same part of each specimen. Furthermore, higher values are often measured in coatings, where gloss is generally measured, as light is reflected multiple times by the surface, which affects the measurement. To prevent this, the samples were placed on a black matte surface where light was not reflected. The reflectivity and glossiness values produced by FIM were slightly higher than those produced with CIM. However, the difference was not found to be significant (Figure 5).

**Figure 6:**
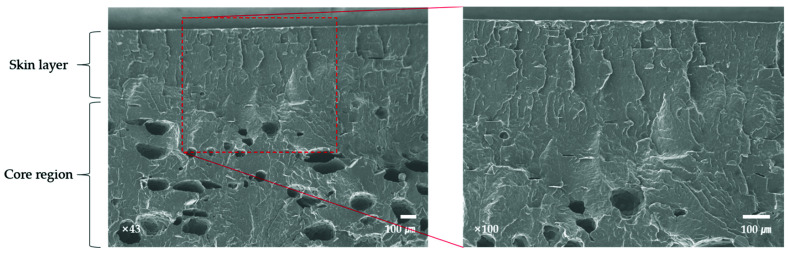
SEM image of aluminum 3% FIM.

A previous study on the normal flow region of aluminum flakes demonstrates that fractional flow causes aluminum flakes to move vertically from the core layer, and most of them are oriented parallel to the skin layer [34]; thus, the remaining fillers in the core layer are believed to have been affected by voids (Figure 6).

### 3.2. Lightweight and Mechanical Properties Evaluation

#### 3.2.1. Foaming Ratio

**Figure 7:**
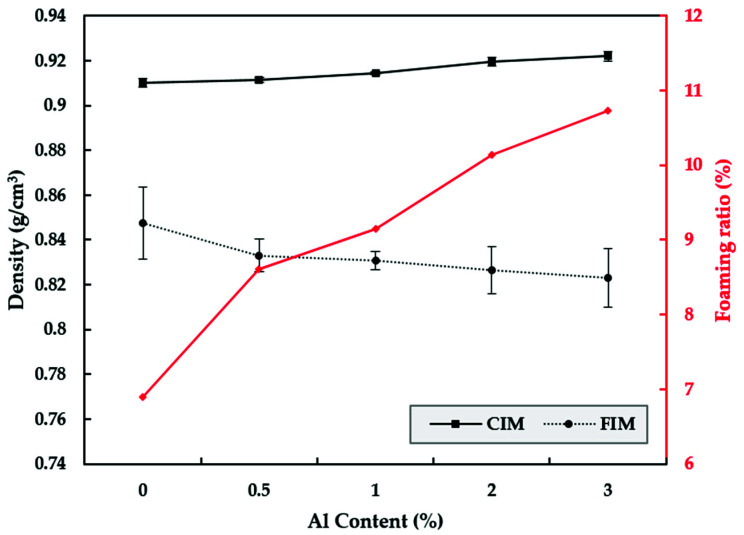
Relationships between density variation and aluminum content and foam rate and aluminum content.

Aluminum is a nonferrous metal that is denser than polymeric resin. Thus, the density of the specimen increases as the content increases and evokes a metal texture (Figure 7). However, the shortcomings of increasing mass can be overcome while maintaining a metallic texture through gas foaming. In addition, the increase of aluminum flake content can cause more foaming when FIM is used, and the extra foam is believed to serve as a crystalline nucleus for inorganic systems such as talc.

The long-studied theory of cell formation by nuclear agents suggests that single-phase dissolved polymers and gases and the low free energy provided by the nuclear interfaces are used for the growth of non-uniform nuclei [35].

#### 3.2.2. Mechanical Properties

**Figure 8:**
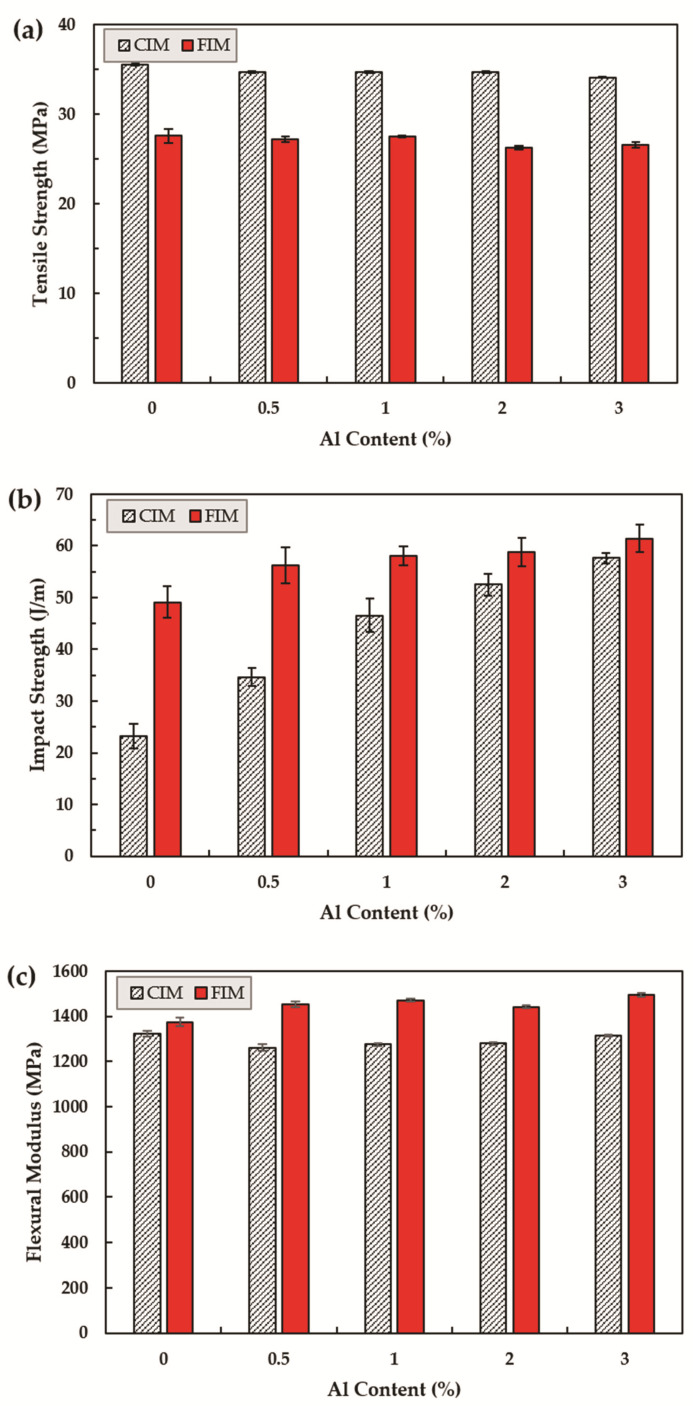
Summary of mechanical properties with different quantities of aluminum flake, ranging from 0 to 3 wt%; (**a**) Tensile strength; (**b**) Impact strength; (**c**) Flexural modulus.

Tensile and impact strength and flexural modulus were independently measured for seven samples, and five mean values were used, after excluding the maximum and minimum values (Figure 8).

The tensile strength tended to decrease as the filling content of aluminum flakes and polypropylene compositions increased. However, the strength of neat PP without the filler was 35.5 MPa, while the strength with 3% added was 34.1 MPa, indicating that the material reduction was insignificant. The flexural modulus also tended to decrease, as did the tensile strength, resulting in a reduction of approximately 40 MPa. The data for this configuration are shown in Figure 5. The above data alone are not sufficient to determine which description is valid. However, a previous study demonstrated that there is no interfacial adhesion between the polymers and fillers, resulting in phase separation. The geometry of the fillers is two-dimensional (flat, thin, and sharp), making them more breakable than other composites [36,37]. Another explanation for the trend in intensity reduction is PP’s crystallinity. It explain why the overall tensile strength has been reduced, because aluminum filler interferes with crystal growth and reduces crystallinity [37].

The impact strength tended to improve as the aluminum content increased. This is also due to crystal behavior; the lowered crystallization serves as a mechanism to slow the rate of cracking by propagating the absorbed shock energy gradually [36].

Next, the results for the specimen obtained with FIM demonstrate that higher aluminum content results in lower tensile strength. The tensile strength was greatly reduced owing to increased voids due to foaming. In contrast, the impact strength increased significantly. Voids generated in different forms on the polymer slowed the crack, and the cell structure that caused weight reduction per unit area were considered in the paper to help improve impact strength [38]. Furthermore, increasing the aluminum flake content can work to reinforce the composite materials as it promotes cell nucleus formation and growth, absorbing more shock energy [39]. This demonstrates that aluminum flakes and cells improve the shock resistance of the composite, while reducing the strength.

## 4. Conclusions

This study examined the changes in the texture of a metal when foam injection molding was used to form a composite containing aluminum flakes. Samples subjected to ordinary injection were compared by metal pigment form, size, and content, and optical properties were assessed using a gloss meter and a spectrophotometer. Aluminum foam injection molding showed up to 7 GU higher gloss than conventional injection method, and the reflectance improved by 6%. The reason for such enhancements was analyzed using SEM. It is also important to identify the mechanical properties that enable plastics to exhibit a metallic texture. Plastics have lower mechanical properties than metals; thus, their impact strength and flexural elasticity were improved by 62% and 15%, respectively, through foaming. However, the tensile strength of 20% decreased owing to the reduced surface adhesion of additives. Aluminum foam injection is an eco-friendly method suitable for replacing existing painting processes. Aluminum-foam-injection-molded products are 11% lighter than conventional plastic products and can be used in a manner similar to metals. Further research on the basic properties of aluminum, such as conductivity and bonding, could facilitate the development of a metal replacement product for the industry.

## Figures and Tables

**Table 1:** Aluminum flakes of different shapes and sizes used in the study.

Grade	TFP 013-30-E1	TFP 032-30-E1	SVT 460-30-E1	SS 960-30-E1
**Type**	Silver Dollar	Silver Dollar	Silver Dollar	Cornflake
**Particle size (μm)**	13	32	65	14
**Specific gravity**	1.72	1.72	1.69	1.72

**Table 2:** Conditional parameters for CIM and FIM.

Parameter		Experimental Conditions of Injection Molding Machine				
		CIM			FIM	
Injection	Temp. (°C)	Nozzle	Heater 1	Heater 2	Heater 3	Heater 4
		220	210	200	190	180
	Pressure (MPa)	7				
	Speed (%)	100				
Gas	Pressure (MPa)	-			4	
	Blowing agent	-			N_2_	
Holding	Pressure (MPa)	6.5, 3.2			-	
	Time (s)	2, 1			-	
Mold	Temp. (°C)	60				
Room Temp. (°C)		21 ± 3				
Cooling	Time (s)	100				

**Table 3:** Comparison of color spaces depending on aluminum flake type.

	Sample 1			Sample 2		
**Grade**	TFP 013-30-E1			SS 960-30-E1		
**Type**	Silver Dollar			Cornflake		
**Particle size**	13 μm			14 μm		
**Color space**	L*	a*	b*	L*	a*	b*
	81.5	−0.45	−1.03	75.8	−0.55	−1.60

The * symbol indicates that this is a new color system in the older CIELAB system. The L* is the lightness value, and a* is the green-red colors, and b* is the blue-yellow opponents.

**Table 4:** Color space comparison for aluminum flakes of different sizes.

	Sample 1			Sample 2			Sample 3		
**Grade**	TFP 013-30-E1			TFP 032-30-E1			SVT 460-30-E1		
**Type**	Silver Dollar								
**Particle size**	13 μm			32 μm			65 μm		
**Color space**	L*	a*	b*	L*	a*	b*	L*	a*	b*
	81.9	−0.4	−0.73	79.2	−0.29	0	70.3	−0.67	−0.02

The * symbol indicates that this is a new color system in the older CIELAB system. The L* is the lightness value, and a* is the green-red colors, and b* is the blue-yellow opponents.

**Table 5:** Comparison of color spaces for products produced by conventional and foam injection methods.

Process		SVT 460-30-E1 (SD Type, 65 μm)							
		CIM				FIM			
**Al Content (%)**		**0**	**1**	**2**	**3**	**0**	**1**	**2**	**3**
**Color space**	L*	38.7	66.7	69.5	70.3	75.3	68.2	69.9	70.8
	a*	−0.63	−0.50	−0.64	−0.67	−0.19	−0.63	−0.68	−0.72
	b*	−4.36	0.34	0.07	−0.02	−0.46	−0.16	−0.16	−0.12

The * symbol indicates that this is a new color system in the older CIELAB system. The L* is the lightness value, and a* is the green-red colors, and b* is the blue-yellow opponents.

## Data Availability

Not applicable.
